# The impact of geriatric nutritional risk index on esophageal squamous cell carcinoma patients with neoadjuvant therapy followed by esophagectomy

**DOI:** 10.3389/fnut.2022.983038

**Published:** 2022-10-20

**Authors:** Pinhao Fang, Qian Yang, Jianfeng Zhou, Yushang Yang, Siyuan Luan, Xin Xiao, Xiaokun Li, Yimin Gu, Qixin Shang, Hanlu Zhang, Longqi Chen, Xiaoxi Zeng, Yong Yuan

**Affiliations:** ^1^Department of Thoracic Surgery, Med+X Center for Informatics, West China Hospital, Sichuan University, Chengdu, China; ^2^Anesthesia Operation Center of West China Hospital, West China School of Nursing, Sichuan University, Chengdu, China; ^3^Biomedical Big Data Center of West China Hospital, Med+X Center for Informatics, Sichuan University, Chengdu, China

**Keywords:** esophageal cancer, neoadjuvant therapy, geriatric nutritional risk index, postoperative complications, prognosis

## Abstract

**Background:**

The Geriatric Nutritional Index (GNRI) has been indicated as a nutritional index which is highly associated with complications and mortality in older hospitalized patients. Moreover, early studies had suggested that GNRI is a potential prognostic indicator for some malignances. However, the prognostic value of GNRI in esophageal squamous cell carcinoma (ESCC) patients underwent neoadjuvant therapy followed by esophagectomy remains elusive.

**Materials and methods:**

This retrospective study incorporated 373 patients with ESCC who had underwent neoadjuvant therapy followed by radical esophagectomy at West China Hospital of Sichuan University between April 2011 and September 2021. The GNRI formula was: 1.489 × albumin (g/dl) + 41.7 × current weight/ideal weight. Patients were classified as GNRI-low (GNRI < 98.7) or GNRI high (GNRI ≥ 98.7). The association between GNRI and clinical survival status were assessed utilizing Kaplan-Meier methods and Cox regression analysis.

**Results:**

Three hundred and seventy three patients were retrospectively included in this study. 80 (21.5%) and 293 (78.5%) patients had been divided into the GNRI-low and GNRI-high groups respectively. Pathological T stage and the rate of nodal metastasis were significantly higher in the GNRI low group than in the GNRI high group (*P* = 0.003 and *P* = 0.001, respectively) among the examined demographic parameters. Furthermore, GNRI was significantly correlated with postoperative complications, patients with lower GNRI had a higher postoperative complication rate as compared with GNRI high group [Odds ratio: 2.023; 95% confidence interval (CI): 1.208–3.389; *P* = 0.007]. Univariate analysis of 5-year overall survival (OS) and disease-free survival (DFS) found that the rate of survival was considerably lower in the GNRI-low group than in the GNRI-high group (*P* < 0.001). However, multivariate analysis demonstrated that GNRI was not an independent risk factor.

**Conclusion:**

In patients with ESCC, low GNRI exhibited a poor nutritional indicator and related to postoperative complications after neoadjuvant therapy. Intensive follow-up after surgery should be performed for ESCC patients with low GNRI.

## Introduction

Esophageal carcinoma (EC) is one of the most aggressive malignant tumors and is also the world’s sixth-leading cause of cancer-related death (1). Squamous cell carcinoma (ESCC) and adenocarcinoma (EAC) are two main pathological types of EC, and ESCC is the most common pathological type. Surgical therapy plays a predominate role in EC treatment and the surgery types are mainly represented by the following: Ivor-Lewis, Mckeown, Sweet and transhiatal esophagectomy (2–4). Though the therapy of EC had advanced rapidly in recent decades (5), the overall 5-year survival rate of EC patients remained unsatisfactory. In patients with advanced localized EC, neoadjuvant therapies such as chemoradiotherapy could downstage the primary tumor and prolong the prognosis of EC patients after surgery (6). Nutritional evaluation and support are important parts during the whole management of cancer. Malnutrition is typically manifested as a low BMI which has been reported to associate with higher postoperative complications rate and a poor prognosis in individuals with benign or malignant diseases (7). Due to the invasive, malignant characteristics and of malignant digestive strictures of EC, patients with EC may experience dysphagia and progress into malnutrition (8).

As a novel nutritional evaluating index, geriatric nutritional index (GNRI) was first reported by Bouillanne et al. (9) in 2005. The exact value of GNRI could be easily calculated from serum albumin level and the ratio of normal body weight to ideal body weight, and GNRI was more closely connected with nutrition-related complications and deaths in older hospitalized patients than BMI and serum albumin level alone (8). Early studies had indicated that GNRI was a potential prognosis indicator in EC patients, and low GNRI could usually lead to reduce the quality of life (10). However, the effect of GNRI on postoperative outcomes in ESCC patients treated with neoadjuvant therapy followed by esophagectomy has not been well-studied. We hypothesized that GNRI was a better predictor of postoperative complications and a more independent prognostic factor in individuals receiving esophagectomy than a low BMI alone. As a result, this study was to investigate the impact of GNRI status on the prognosis for patients with ESCC.

## Materials and methods

### Study patients

All the ESCC patients included in had been treated with neoadjuvant therapy and followed by esophagectomy. The criteria for inclusion and exclusion were as follows: (1) patients were pathological diagnosed as ESCC; (2) patients had underwent esophagectomy resection; (3) patients had been treated with neoadjuvant therapy before esophagectomy; (4) patients had been followed-up enough time. Exclusion criteria were: (1) patients with distant tumor metastases; (2) patients treated with chemoradiotherapy after surgery; (3) patients underwent immunotherapy. Overall survival (OS) and disease-free survival (DFS) was selected as the duration from primary operation to death or tumor recurrence. Three hundred and seventy three patients pathologically diagnosed as ESCC underwent esophagectomy were included in this retrospective analysis at West China Hospital, Sichuan University.

#### Patient’s therapy

Patients with locally advanced ESCC (T2-T4 or N1-3 M0) had received the neoadjuvant therapy before surgery according to the guideline (11). Neoadjuvant therapy was administered to patients in accordance with national recommendations. In general, the neoadjuvant chemotherapy regimen involved two cycles, with a 3 week break between each cycle. All patients received paclitaxel (175 mg/m^2^ body-surface area, D1) and cisplatin (75 mg/m^2^ body-surface area, D1) intravenously over through the period of two cycles. As the aspect of neoadjuvant chemoradiotherapy regimen, all patients received a total radiation dosage of 40–50.4 Gy in 23–28 fractions (1.8–2.0 Gy/fraction), two cycles of the simultaneous chemotherapy drugs paclitaxel (175 mg/m^2^ body-surface area, D1, q3w) and cisplatin (75 mg/m^2^ body-surface area, D1, q3w). Intensity-modulated radiotherapy was used to provide radiation to all the patients. Surgery for surgical resection was carried out using the typical McKeown, Ivor-Lewis and Sweet methods 6–8 weeks following the end of neoadjuvant treatment. All patients had standard two-field (abdominal and thoracic) lymph node excision. Three-field lymph node dissection was not commonly used in the research, and cervical lymph node dissection was selected for patients with suspicious cervical lymph node metastases as determined by preoperative CT and ultrasound. Detailed surgical techniques have already been documented (9, 12). All patients in the research cohort were followed until death or September 2021, whichever occurred first. For the first 5 years after surgery, all patients had neck, abdomen, and thoracic computed tomography scans and biochemical blood tests every 4 months, as well as an endoscopy every year. Overall survival (OS) was calculated from the day of operation to September 2021 or until death was confirmed. Disease-free survival (DFS) was assessed from the day of surgery to the day of cancer recurrence, death, or September 2021.

### Index calculation

The assessment of GNRI in all patients was performed during the period after neoadjuvant therapy and before esophagectomy. Based on the results from the X-tile program, the optimal cutoff points for overall survival were determined to be 98.7 (Supplementary Figure 1). The GNRI was calculated as follows: GNRI = 1.489 × albumin (g/dl) + 41.7 × usual weight/ideal weight. The Lorentz formula calculated ideal weight: ideal weight = 22 × height (m) × height (m). The total GNRI score was classified as no risk (GNRI ≥ 98.7) or risk (GNRI < 98.7) of malnutrition.

The Union for International Cancer Control TNM Classification of Malignant Tumors (8th edition) was used for pathological diagnosis and disease classification (13). The Clavien Dindo classification was used to assess the severity of postoperative complications (14). The postoperative complication was defined in this study as the presence of grade II complications according to the Clavien Dindo grading system (14). All the patient characteristics were collected from their medical and nursing records. The ethics committee of West China Hospital, Sichuan University, authorized this study. All patients gave their informed permission.

### Statistics analysis

The Mann-Whitney’s U test was used to compare continuous variables, whereas, the Fisher’s exact test was used to compare categorical variables. The risk variables for postoperative complications were evaluated using logistic multivariate analysis. The Kaplan-Meier method was used to calculate OS and DFS within subgroups, and the log-rank test was applied to compare prognoses between groups. For univariate and multivariate analysis, the Cox proportional hazards model was utilized to identify independent prognostic indicators for OS and DFS. *P* < 0.05 represented statistical significance. For all statistical analyses, the SPSS software (version 26.0; SPSS) was utilized.

## Results

### Clinicopathological characteristics according to geriatric nutritional index

Three hundred and five males and 68 females met the inclusion criteria and were finally incorporated into analysis. The tumor was found in the middle thoracic esophagus in 61.7% (230/373) of the cases, while nodal metastasis was found in 35.4% (132/373) of the patients. Overall, all patients had neoadjuvant chemotherapy or chemoradiotherapy. The 5-year OS and DFS rates for the entire cohort were 66.6% and 58.5%, respectively.

The characteristics of patients stratified by GNRI risk are shown in Table 1. In summary, 80 (21.5%) and 293 (78.5%) patients were in the GNRI-low and GNRI-high group respectively. No statistically significant differences were found in age or gender between the two groups. BMI was significantly lower in the GNRI-low group than in the GNRI-high group (*P* < 0.001). The pathological T stage and rate of nodal metastasis in the GNRI-low group were markedly higher than in the GNRI-high group (*P* = 0.003 and *P* = 0.001, respectively).

**TABLE 1:** Patient characteristics and geriatric nutritional index (GNRI).

	Cases	GNRI		*P-value*
	(*n* = 373)	Low [*N* = 80] (21.5%)	High [*N* = 293] (78.5%)	
**Sex**				
Male	305	67 (83.3%)	238 (81.2%)	0.605
Female	68	13 (16.3%)	55 (18.8%)	
**Age**				
<60	149	25 (31.3%)	124 (42.3%)	0.073
≥60	224	55 (68.8%)	169 (57.7%)	
**BMI**				
<18.5	42	37 (46.3%)	5 (1.7%)	**<0.001**
≥18.5	331	43 (53.8%)	288 (98.3%)	
**Localization**				
Upper	45	11 (13.8%)	34 (11.6%)	0.313
Middle	230	44 (55.0%)	186 (63.5%)	
Lower	96	24 (30.0%)	72 (24.6%)	
Gastroesophageal junction	2	1 (1.3%)	1 (0.3%)	
**Pathological T stage**				
pT0, 1, 2	248	42 (52.5%)	206 (70.3%)	**0.003**
pT3, 4	125	38 (47.5%)	87 (29.7%)	
**Pathological N stage**				
N negative	241	39 (48.8%)	202 (68.9%)	**0.001**
N positive	132	41 (51.2%)	91 (31.1%)	
**Differentiation grade**				
Well	170	27 (33.8%)	143 (48.8%)	0.056
Moderated	94	24 (30.0%)	70 (23.9%)	
Poor	109	29 (36.2%)	80 (27.3%)	
**Tumor length, cm**				
<3	238	38 (47.5%)	200 (68.3%)	**0.001**
≥3	135	42 (52.5%)	93 (31.7%)	
**Surgery type**				
Sweet	16	5 (6.3%)	11 (3.8%)	0.527
Ivor-Lewis	228	49 (61.3%)	179 (61.1%)	
Mckeown	129	26 (32.5%)	103 (35.2%)	
**Preoperative treatment**				
Neoadjuvant chemoradiotherapy	296	62 (77.5%)	234 (79.9%)	0.643
Neoadjuvant chemotherapy	77	18 (22.5%)	59 (20.1%)	
**TRS**				
TRS = 0, 1	197	33 (41.3%)	164 (56.0%)	**0.019**
TRS = 2, 3	176	47 (58.8%)	129 (44.0%)	

BMI, body mass index; GNRI, geriatric nutritional index; TRS, tumor regression scoring. The bold values indicated the *P*-value lower than 0.05 with statistical differences.

### Geriatric nutritional index and short- and long-term outcomes of curative surgery following neoadjuvant therapy

**FIGURE 1:**
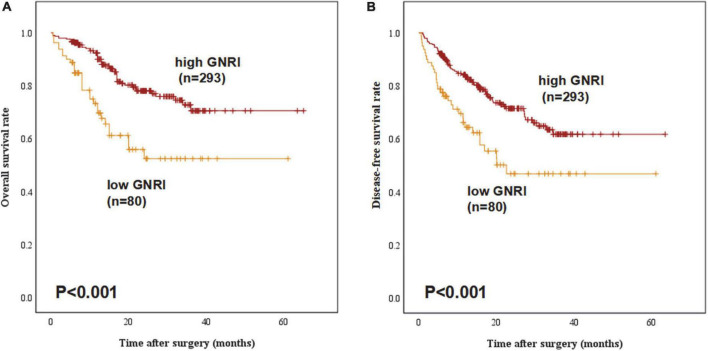
Overall survival and disease-free survival curves stratified by geriatric nutritional index (GNRI) status: **(A)** overall survival and **(B)** disease-free survival of all study patients (*n* = 373).

Figure 1 demonstrated both groups’ Kaplan Meier curves for OS and DFS based on GNRI group. In summary, the 5-year OS and DFS rates in the GNRI-low group were 52.3% and 46.7%, respectively, substantially lower than the GNRI-high group (70.5% and 61.7%, *P* < 0.001 and *P* < 0.001, respectively). Patients in the GNRI-low group had a higher 90-day mortality rate (8.8%) after surgery compared with those in GNRI-high group (2.0%, *P* = 0.009). Table 2 demonstrated the association between GNRI, another conventional nutritional index BMI and the correlated postoperative complication rate. The postoperative complications rate was significantly higher in the GNRI-low group than in the GNRI-high group. Except anastomotic leakage [14.3% (6/42) vs. 5.1% (17/331), *P* = 0.020], no significant differences were found in BMI between the two groups. Table 3 showed the results of logistic regression analysis used to investigate risk variables for postoperative complications. The univariate analyses results showed that GNRI was a risk factor of postoperative complications [Odds ratio (OR), 2.037; 95% confidence interval (CI), 1.219–3.404; *P* = 0.007]. Additionally, the results of multivariate analysis demonstrated that GNRI was an independent predictor of postoperative complications (OR, 2.023; 95% confidence interval CI, 1.208–3.389; *P* = 0.007). However, BMI was proved not associated with the postoperative complications (OR, 1.689; 95% CI, 0.873–3.270; *P* = 0.120).

**TABLE 2:** Postoperative complications stratified by geriatric nutritional index (GNRI), the level of serum albumin, and body mass index (BMI) value.

	Cases (*n* = 373)	GNRI-low (*N* = 80)	GNRI-high (*N* = 293)	*P-value*
Lung complication	111	34 (42.5%)	77 (26.3%)	**0.005**
Anastomotic leakage	23	10 (12.5%)	13 (4.4%)	**0.008**
Pleural effusion	56	21 (26.3%)	35 (11.9%)	**0.002**

	**Cases (*n* = 373)**	**BMI-low (*N* = 42)**	**BMI-high (*N* = 331)**	***P-value***

Lung complication	111	17 (40.5%)	94 (28.4%)	0.107
Anastomotic leakage	23	6 (14.3%)	17 (5.1%)	**0.020**
Pleural effusion	56	9 (21.4%)	47 (14.2%)	0.217

BMI, body mass index; GNRI, geriatric nutritional index.

**TABLE 3:** Logistic regression analysis for clinical factors associated with complications after surgery.

Factors	Univariate analysis		Multivariate analysis	
	OR (95% CI)	*P-value*	OR (95% CI)	*P-value*
Sex (male/female)	1.826 (0.968–3.446)	0.063	1.806 (0.952–3.426)	0.070
Age (≥60/<60)	1.441 (0.908–2.287)	0.121		
Smoke (yes/no)	0.901 (0.578–1.403)	0.643		
Coronary artery disease (present/absent)	1.076 (0.330–3.505)	0.904		
Hypertension (present/absent)	1.217 (0.654–2.263)	0.536		
Preoperative treatment (nCRT/nCT)	0.973 (0.573–1.713)	0.973		
BMI (low/high)	1.689 (0.873–3.270)	0.120		
GNRI (low/high)	2.037 (1.219–3.404)	**0.007**	2.023 (1.208–3.389)	**0.007**

BMI, body mass index; GNRI, geriatric nutritional index; nCRT, neoadjuvant chemoradiotherapy; nCT, neoadjuvant chemotherapy. The bold values indicated the *P*-value lower than 0.05 with statistical differences.

According to the univariate analysis, sex [Hazard ratio (HR), 2.686; 95% CI, 1.235–5.841; *P* = 0.013], tumor length (HR, 1.906; 95% CI, 1.219–2.981; *P* = 0.005), pT (HR, 2.830; 95% CI, 1.809–4.429; *P* < 0.001), pN (HR, 4.056; 95% CI, 2.549–6.455; *P* < 0.001), tumor differentiation grade (HR, 1.616; 95% CI, 1.243–2.100; *P* < 0.001), tumor regression scoring (TRS) (HR, 2.576; 95% CI, 1.601–4.147; *P* < 0.001), BMI (HR, 2.650; 95% CI, 1.547–4.540; *P* < 0.001) and GNRI (HR, 2.601; 95% CI, 1.635–4.137; *P* < 0.001) significantly affected the OS of ESCC patients (Table 4A). However, the multivariate analysis results showed that GNRI (HR, 1.678; 95% CI, 0.916–3.075; *P* = 0.094) or BMI (HR, 1.193; 95% CI, 0.575–2.474; *P* = 0.636) were not independent prognostic factor of OS (Table 4B).

**TABLE 4A:** Univariate analysis of prognostic factors associated with overall survival.

Factors	Univariate analysis	
	HR (95% CI)	*P-value*
Sex (male/female)	2.686 (1.235–5.841)	**0.013**
Age (≥60/<60)	0.989 (0.630–1.554)	0.963
Tumor length (≥3/<3 cm)	1.906 (1.219–2.981)	**0.005**
Localization	0.849 (0.588–1.227)	0.384
pT stage	2.830 (1.809–4.429)	**<0.001**
pNstage	4.056 (2.549–6.455)	**<0.001**
Differentiation grade	1.616 (1.243–2.100)	**<0.001**
Preoperative treatment (nCRT/nCT)	1.184 (0.691–2.029)	0.539
TRS (2, 3/0, 1)	2.576 (1.601–4.147)	**<0.001**
BMI (<18.5/≥18.5)	2.650 (1.547–4.540)	**<0.001**
GNRI (low/high)	2.601 (1.635–4.137)	**<0.001**

TRS, tumor regression scoring; BMI, body mass index; GNRI, geriatric nutritional index; nCRT, neoadjuvant chemoradiotherapy; nCT, neoadjuvant chemotherapy. The bold values indicated the *P*-value lower than 0.05 with statistical differences.

**TABLE 4B:** Multivariate analysis of prognostic factors associated with overall survival.

Factors	Multivariate analysis	
	HR (95% CI)	*P-value*
Sex (male/female)	1.887 (0.854–4.167)	0.116
Tumor length (≥3/<3 cm)	1.384 (0.863–2.220)	0.177
pT stage	1.549 (0.784–3.057)	0.208
pN stage	2.791 (1.679–4.639)	**<0.001**
Differentiation grade	1.088 (0.745–1.589)	0.663
TRS (2,3/0,1)	0.973 (0.443–2.136)	0.945
BMI (<18.5/≥18.5)	1.193 (0.575–2.474)	0.636
GNRI (low/high)	1.678 (0.916–3.075)	0.094

TRS, tumor regression scoring; BMI, body mass index; GNRI, geriatric nutritional index. The bold values indicated the *P*-value lower than 0.05 with statistical differences.

Additionally, the univariate analysis also demonstrated that sex (HR, 2.025; 95% CI, 1.109–3.695; *P* = 0.020), tumor length (HR, 1.702; 95% CI, 1.160–2.497; *P* = 0.007), pT (HR, 2.737; 95% CI, 1.864–4.019; *P* < 0.001), pN (HR, 3.175; 95% CI, 2.151–4.687; *P* < 0.001), tumor differentiation grade (HR, 1.565; 95% CI, 1.250–1.961; *P* < 0.001), TRS (HR, 2.303; 95% CI, 1.543–3.438; *P* < 0.001), BMI (HR, 2.292; 95% CI, 1.407–3.733; *P* = 0.001) and GNRI (HR, 2.101; 95% CI, 1.390–3.175; *P* < 0.001) were significantly correlated with DFS of ESCC patients (Table 5A). Nevertheless, GNRI was indicated not to be an independent prognostic factor through multivariate analysis (HR, 1.438; 95% CI, 0.848–2.440; *P* = 0.178), and the results of multivariate analysis also demonstrated that lower BMI was not associated with poorer DFS (HR, 1.290; 95% CI, 0.681–2.446; *P* = 0.435), whereas pT (HR, 1.810; 95% CI, 1.008–3.252; *P* = 0.047) and pN (HR, 2.322; 95% CI, 1.524–3.538; *P* < 0.001) stage were proved to be independent prognosis factors (Table 5B).

**TABLE 5A:** Univariate analysis of prognostic factors associated with disease-free survival.

Factors	Univariate analysis	
	HR (95% CI)	*P-value*
Sex (male/female)	2.025 (1.109–3.695)	**0.020**
Age (≥60/<60)	0.992 (0.672–1.464)	0.969
Tumor length (≥3/<3 cm)	1.702 (1.160–2.497)	**0.007**
Localization	0.867 (0.634–1.187)	0.374
pT stage	2.737 (1.864–4.019)	**<0.001**
pNstage	3.175 (2.151–4.687)	**<0.001**
Differentiation grade	1.565 (1.250–1.961)	**<0.001**
Preoperative treatment (nCRT/nCT)	1.067 (0.667–1.708)	0.786
TRS (2,3/0,1)	2.303 (1.543–3.438)	**<0.001**
BMI (<18.5/≥18.5)	2.292 (1.407–3.733)	**0.001**
GNRI (low/high)	2.101 (1.390–3.175)	**<0.001**

TRS, tumor regression scoring; BMI, body mass index; GNRI, geriatric nutritional index; nCRT, neoadjuvant chemoradiotherapy; nCT, neoadjuvant chemotherapy. The bold values indicated the *P*-value lower than 0.05 with statistical differences.

**TABLE 5B:** Multivariate analysis of prognostic factors associated with disease-free survival.

Factors	Multivariate analysis	
	HR (95% CI)	*P-value*
Sex (male/female)	1.500 (0.812–2.771)	0.195
Tumor length (≥3/<3 cm)	1.310 (0.875–1.960)	0.189
pT stage	1.810 (1.008–3.252)	**0.047**
pN stage	2.322 (1.524–3.538)	**<0.001**
Differentiation grade	1.109 (0.807–1.524)	0.525
TRS (2,3/0,1)	0.888 (0.457–1.724)	0.725
BMI (<18.5/≥18.5)	1.290 (0.681–2.446)	0.435
GNRI (low/high)	1.438 (0.848–2.440)	0.178

TRS, tumor regression scoring; BMI, body mass index; GNRI, geriatric nutritional index. The bold values indicated the *P*-value lower than 0.05 with statistical differences.

### Geriatric nutritional index is a prognostic indicator for esophageal squamous cell carcinoma patients with normal body mass index

**FIGURE 2:**
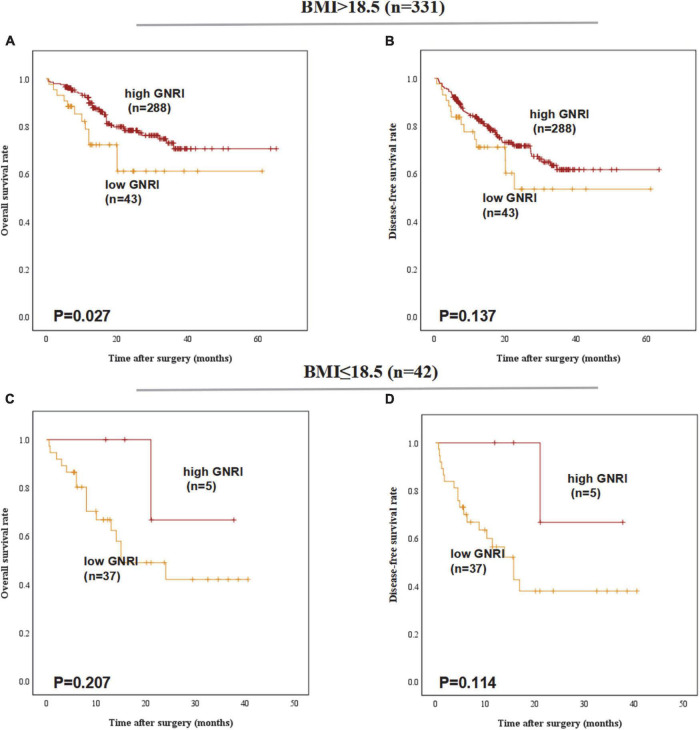
Postoperative outcomes of patients with high or low geriatric nutritional index (GNRI) according to BMI: **(A)** overall survival, **(B)** disease-free survival of BMI > 18.5 patients (*n* = 331), **(C)** overall survival, and **(D)** disease-free survival of BMI ≤ 18.5 patients (*n* = 42).

A comparison of 5-year OS and DFS rates between the GNRI-high and GNRI-low groups that were stratified according to BMI revealed that only among patients with BMI ≥ 18.5, 5-year OS was significantly worse in the GNRI-low group than in the GNRI-high group (61.1% vs. 70.6%, *P* = 0.027). In contrast, no significant differences were noted between the two groups among patients with BMI < 18.5 (42.0% vs. 66.7%, *P* = 0.207) and no significant differences were found in DFS (Figure 2).

### Geriatric nutritional index is a prognostic indicator for patients underwent various types of esophagectomy

**FIGURE 3:**
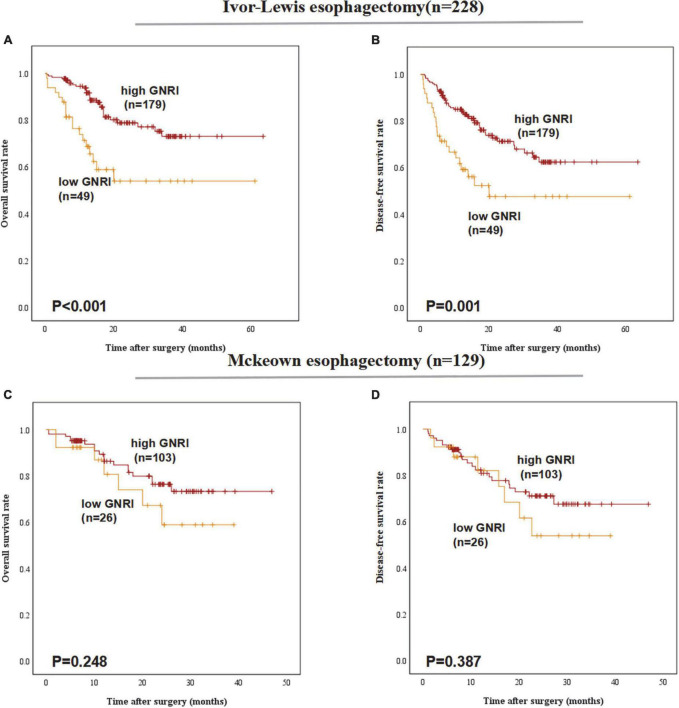
Postoperative outcomes of patients with high or low geriatric nutritional index (GNRI) basing on different surgery types: **(A)** overall survival, **(B)** disease-free survival of esophageal squamous cell carcinoma (ESCC) patients underwent Ivor-Lewis esophagectomy (*n* = 228), **(C)** overall survival, and **(D)** disease-free survival of ESCC patients underwent Mckeown esophagectomy (*n* = 129).

In the group of different esophagectomy types, low-GNRI was proven to be a worse predictor for the OS (*P* < 0.001) and DFS (*P* = 0.001) in patients underwent Ivor-Lewis esophagectomy. In ESCC patients underwent Mckeown esophagectomy, low-GNRI was demonstrated not associated with poorer OS (*P* = 0.248) nor DFS (*P* = 0.387) (Figure 3).

### Geriatric nutritional index is a prognostic indicator of overall survival for patients underwent different preoperative treatments

**FIGURE 4:**
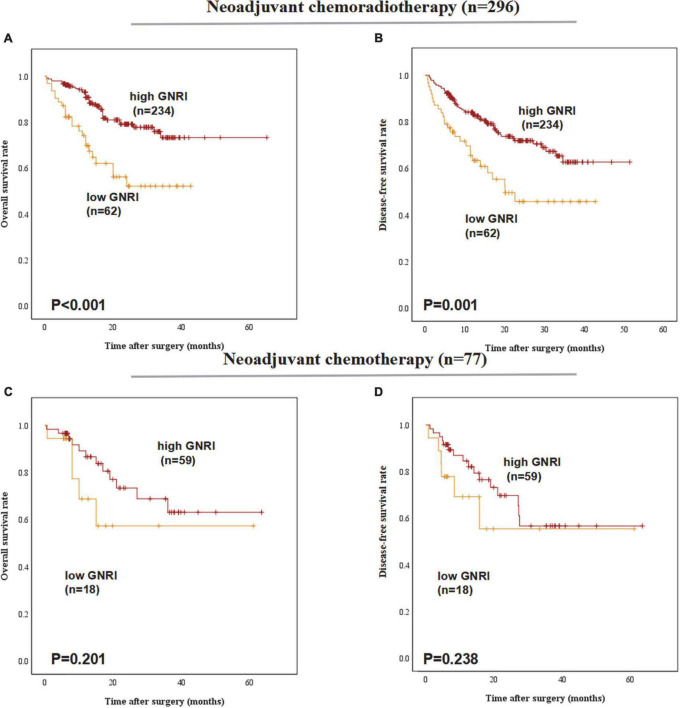
Postoperative outcomes of patients with high or low geriatric nutritional index (GNRI) basing on different preoperative treatment: **(A)** overall survival, **(B)** disease-free survival of ESCC patients underwent neoadjuvant chemoradiotherapy (*n* = 296), **(C)** overall survival, and **(D)** disease-free survival of ESCC patients underwent neoadjuvant chemotherapy (*n* = 77).

In the subgroup of preoperative treatments, low-GNRI was proven to be a poorer predictor for the OS (*P* < 0.001) and DFS (*P* = 0.001) in ESCC patients underwent neoadjuvant chemoradiotherapy before esophagectomy. But in patients with neoadjuvant chemotherapy preoperatively, low-GNRI was proved not associated with worse OS (*P* = 0.201) nor DFS (*P* = 0.238) (Figure 4).

## Discussion

Bouillanne et al. had first proposed using GNRI as a risk index to evaluate nutritional status in elderly patients. They had shown that GNRI is an objective and simple parameter which could be calculated through routine clinical measurement (9). In our study, we discovered that GNRI was an independent predictor of the postoperative complications in patients with ESCC treated with neoadjuvant therapy followed by esophagectomy. In EC patients, convention nutritional index such as BMI had been evaluated and was proved to associated with EC prognosis before. As a novel index to measure the nutritional level, GNRI is a simple objective nutritional evaluation score calculated through serum albumin levels and body weight. GNRI has been proven to have clinical relevance as a nutritional morbidity and mortality evaluation tool for older hospitalized patients, as well as those with cardiovascular disease (15), hemodialysis (16), and chronic renal failure (17). However, few investigations have explored the use of GNRI in cancer patients. According to Shoji et al. preoperative GNRI was a predictive factor in older patients with non-small cell lung cancer (18). Some studies have explored the correlation between GNRI and surgical outcomes in EC: Bo et al. found that GNRI was an independent predictive factor for OS in elderly EC patients who underwent radiotherapy (19). Furthermore, Kubo et al. proposed that GNRI was not an independent risk factor for developing pulmonary complications in patients with stage III ESCC but was strongly connected with long-term survival following curative surgery (20). These studies illustrated that preoperative nutritional level associated with the prognosis of patients after surgery and indicated that intervention might ameliorate malnutrition to improve the surgical outcomes of individuals with low GNRI. Few studies had been conducted to determine whether GNRI impacts complications and long-term prognosis in ESCC patients after neoadjuvant treatment. To our knowledge, among all studies investigating the prognostic value of GNRI in ESCC, the sample size in our study is the largest and we had also detected the impact of GNRI on the DFS to gain a more comprehensive understanding of GNRI on survival outcomes of ESCC patients. In addition, all the ESCC patients incorporated into analysis of our study had underwent neoadjuvant therapy and followed by surgery, which made the research patients in our study more specific and more targeted. Furthermore, we had also conducted subgroup analysis to investigate the prognostic value of GNRI in depth basing on the conventional nutritional index BMI, different surgery types and preoperative therapies.

By univariate analysis, there was a significant connection between low GNRI and poor survival in the current research. Especially in group of BMI higher than 18.5, patients with GNRI below 98.7 were related to a considerably higher likelihood of poorer OS than those patients with higher GNRI, which indicated that in EC patients with normal BMI, GNRI is a sensitive parameter to predict EC patients with or without better prognosis when treated with neoadjuvant therapy followed by esophagectomy. In multivariate analysis, however, GNRI was not an independent prognostic factor. Furthermore, the subgroup analysis basing on the surgery types showed that in patients underwent Ivor-Lewis esophagectomy, low-GNRI was a poor indicator for both OS and DFS. However, in the group of Mckeown esophagectomy, such significances were not detected. Jezerskyte et al. (21) had conducted a clinical research, the study results showed that EC patients underwent McKeown esophagectomy were more likely to have eating problems such as: vomit, appetite loss and dysphagia compared with those underwent Ivor-Lewis esophagectomy, which might partly account for such discrepancy among survival outcomes. For the 16 patients treated with Sweet esophagectomy, the sample size was too small to get a convincing conclusion, thus the subgroup analysis results need to be further verified by expanding the sample size, and more large-cohort and multi-center studies are needed better to assess the correlation between GNRI and postoperative survival. On the other hand, low-GNRI was shown to be a robust predictor of survival outcomes in patients treated by neoadjuvant chemoradiotherapy.

We evaluated the reliabilities of GNRI as a risk factor for postoperative complications compared with conventional nutritional index BMI. In short term, GNRI was an independent predictor of postoperative complication rate after neoadjuvant treatment, whereas BMI was proved had no significant association on complications rates after surgery according to the results of multivariate analysis. Since the GNRI was based on the serum level of albumin, and GNRI was considered as a marker which can reflect nutritional status. The level of serum albumin was a sensitive and valuable indicator which can indicate malnutrition in EC patients. Low albumin level had been proved to associated with worse survival in patients with various cancer (22). GNRI, that consisted by combination of both serum albumin and body weight might be one valuable nutritional parameter. An effective nutritional assessment tool should be low-cost, simple, calculated through available data and convenient to use. GNRI can be easily calculated by routine clinical test, and the prognostic prediction value of GNRI had been proved by previous studies which was superior to serum albumin and BMI alone (23). According to the results of our study, GNRI might be a superior index compared with BMI which was similar to findings in early studies, and the univariate analysis indicated that low-GNRI was associated with poorer survival outcomes of ESCC patients. However, either low-BMI or low-GNRI was shown to have no significant association with OS or DFS according to the results of multivariate analysis, which suggested that other nutritional status evaluating indexes are in need to predict long-time survival outcomes.

The results of our study suggested that GNRI could be utilized in clinical setting in the future for confirming ESCC patients with decreasing nutrition level and for patients who requiring nutritional support before esophagectomy. Przekop et al. (24) had proved that GNRI could provide useful prognostic information in patients with head and neck cancer patients qualified for home enteral nutrition (HEN), and they had also suggested nutritional management should be also initiated earlier during the management of cancer patients. Liu et al. (25) demonstrated that HEN and preoperative nutritional support was safe, and beneficial to the recovery of EC patients who had underwent esophagectomy. Therefore, combining the results of GNRI and nutritional support in EC patients during the whole treatment progression seems feasible. It was suggested that in EC patients with low GNRI, providing them with required energy and protein through oral or jejunostomy feeding preoperatively might reduce the postoperative complications rates. Additionally, nutritional support after esophagectomy such as HEN for EC patients may also ameliorate their survival outcomes.

In our study, ESCC patients with lower GNRI were proved to associated subsequent complications, some potential reasons could partly explain the reason. The wound healing after esophagectomy needed adequate energy and nutritional support during the progression of proliferation. Sufficient nutrition supply is of great necessity during the whole progression of EC patients’ management. After neoadjuvant therapy, the swallowing and oral feeding function of EC patients were decreased to some degree because of the side-effects of chemoradiation. Thus, some EC patients were likely to get insufficient nutritional support and progressed into malnutrition. However, malnutrition was a chronic state involving various physiological activities and was difficult to be capture reliably (26). Unlike traditional nutrition evaluating index, the GNRI considered not only the weight of patients, but also the ideal weight and albumin level in peripheral blood and it made GNRI become a screening tool for evaluating nutritional status (27). Previous studies had also found a correlation between malnutrition and immune suppression in cancer patients, leading to postoperative complications and cancer recurrence after surgery (28). Up to now, the main mechanism involving in the relationship between low GNRI and postoperative complications in EC patients following neoadjuvant treatment remains unknown. More molecular biology studies are needed to determine the specific molecular mechanism between malnutrition and postoperative complications in EC patients.

This is a retrospective study assessing the ability of GNRI to predict surgical outcomes in a single, high-volume institute. Notwithstanding, the current study was retrospective in design with all the inherent limitations of such studies. Patients were treated with different dose of radiation or chemotherapy before esophagectomy. Such discrepancy might lead the results of our study deviate from the truth to some extent. Finally, the exact GNRI cutoff value had not reached on consensus which might make it hard to determine the optimal GNRI value in evaluating clinical nutritional status of EC patients. Therefore, more extensive prospective studies involving multiple institutions are warranted in the future.

In conclusion, GNRI was found to be an independent predictive factor of postoperative complications for ESCC patients underwent neoadjuvant therapy followed by surgery. Intensive follow-up nutritional support before surgery should be performed for ESCC patients with low GNRI.

## Data availability statement

The original contributions presented in this study are included in the article/Supplementary material, further inquiries can be directed to the corresponding author.

## Ethics statement

Ethical review and approval was not required for the study on human participants in accordance with the local legislation and institutional requirements. Written informed consent for participation was not required for this study in accordance with the national legislation and the institutional requirements.

## Author contributions

YYu conceptualized the study and revised and proofed the manuscript. PF, QY, and JZ conceptualized the study drafted and proofed the manuscript. YYa, SL, XX, XL, YG, QS, HZ, LC, and XZ collected the literature. All authors contributed to the manuscript revision and revised it critically for intellectual content.

## Supplementary material

Supplementary Figure 1 Kaplan–Meier survival curve stratified by optimal cutoff points in patients with decreased geriatric nutritional index (GNRI) by X-tile software.
