# Supplementary figures and images for: The impact of geriatric nutritional risk index on esophageal squamous cell carcinoma patients with neoadjuvant therapy followed by esophagectomy
Source: Front Nutr. 2022 Oct 20;9:983038. doi: 10.3389/fnut.2022.983038 (PMC9631310; doi:10.3389/fnut.2022.983038)

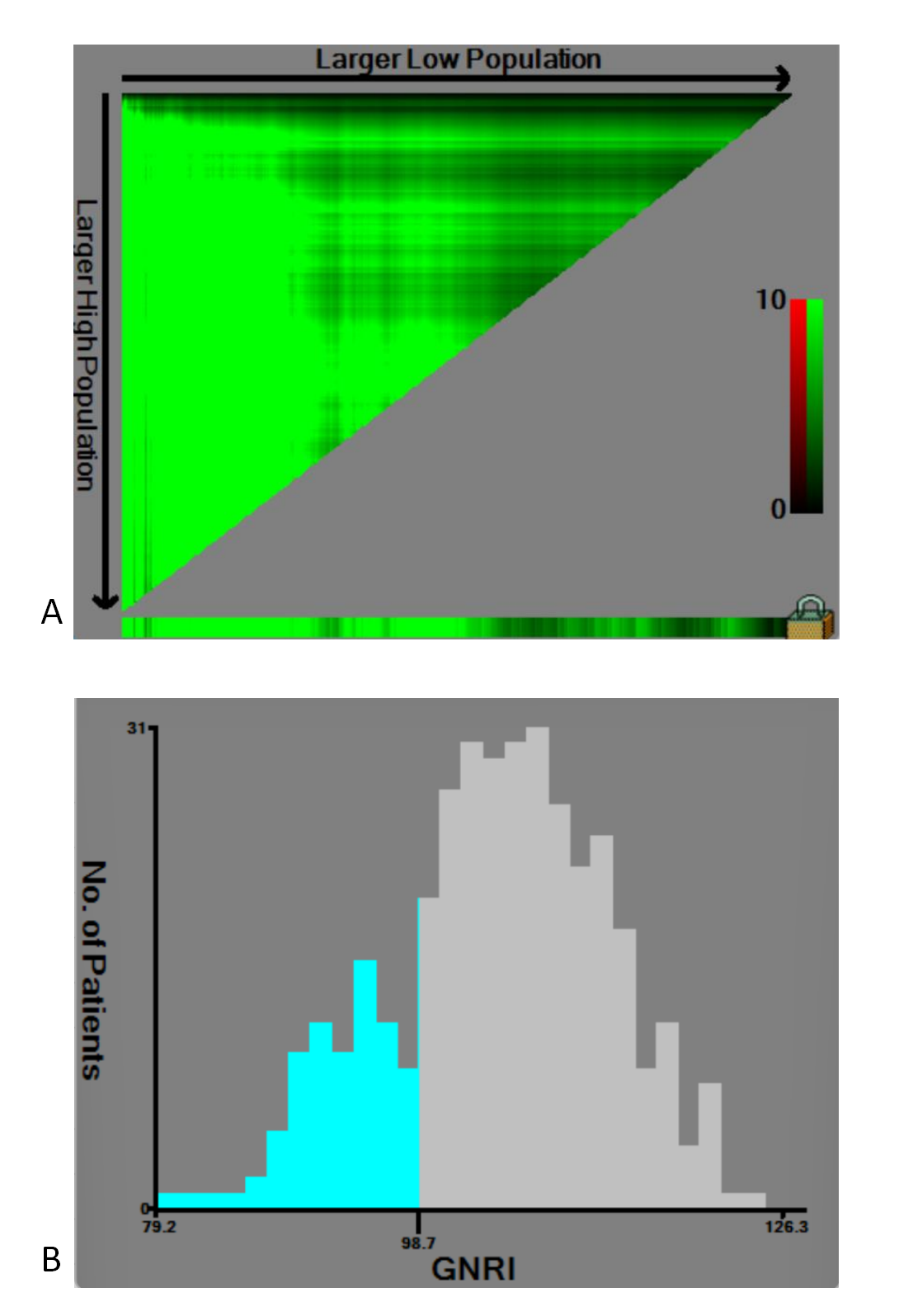

Supplement: Supplementary Figure 1 — Kaplan–Meier survival curve stratified by optimal cutoff points in patients with decreased geriatric nutritional index (GNRI) by X-tile software. [file Image_1.TIF]
